# On the Transmissibility of Syphilis

**Published:** 1855-02

**Authors:** 


On the Transmissibility of Syphilis. By M. Cullerier.—The above author has come to the conclusion that syphilis is not transmissible from the father, but is only manifested in the fœtus when the mother has been infected. If we examine rigorously the case in which the father is supposed to have been the sole cause of hereditary syphilis, we shall find that, at the period of conception, he has transferred the contagion to the mother, who has then directly infected her offspring. Among other illustrative cases M. Cullerier cites the following: A man affected with secondary syphilis married before he was completely cured, his wife immediately became pregnant, and the child which she bore is now eight years old, and the elder brother of two other children, which are in the enjoyment of as vigorous health as itself. In another case, conception occurred simultaneously with the development of secondary syphilis in the father; yet the child which was born is now six years old, and has always enjoyed the most robust health.

M. Vidal has related a case in which a healthy woman, on being united to a man having secondary syphilis, bore a child which immediately died of syphilis. Four years thereafter she married a healthy man, but the child of which he was the father was also syphilitic. Hence Vidal concludes that the semen makes a specific impression on the ovaries, which not only exerts an influence on the products of the primary conception, but also on those of every succeeding one. Cullerier remarks, first of all, that Vidal was able to adduce no proof that the woman, in the case he cited, was free from syphilis. Vidal has adduced instances (similar to those given in Dr. Harvey's excellent papers in *The Monthly Journal*, x. xi.), in which the first conception in animals influenced the product of subsequent intercourse. But these Cullerier does not consider of much importance as being *physiological*, not *pathological* facts. In fine, he considers that the hereditary nature of syphilis is incontestable, but that it is always due to maternal and never to paternal influence. The development of disease in the infant may occur at all periods of fœtal life, and at all periods of maternal infection, either during the existence of an infectious chancre, during the presence of secondary or tertiary symptoms, during the interval of these constitutional manifestations, or even when the mother appears in most perfect health. Hereditary syphilis generally manifests itselt *during the first year of life*, and Cullerier avers that when the infant of infected parents has passed this period, it may be considered to be exempt from the disease. Many authors have maintained an opposite opinion, and believed in the manifestation of hereditary syphilis late in life. This is merely a convenient doctrine. says our author, devised to shield the compromised morality of certain persons. Thus an old author, Jacques de Béthencourt, who wrote in 1527, said that when he met with syphilis among monks and nuns, he always had the charity to believe that it was due to hereditary transmission!—*Archiv. Gen. de Med.*

